# Taylor's Expansion for Composite Functions

**DOI:** 10.1155/2013/536280

**Published:** 2013-09-25

**Authors:** Le Thi Phuong Ngoc, Nguyen Anh Triet

**Affiliations:** ^1^Nhatrang Educational College, 01 Nguyen Chanh Street, Nhatrang City, Vietnam; ^2^Department of Mathematics, University of Architecture of HoChiMinh City, 196 Pasteur Street, District 3, HoChiMinh City, Vietnam

## Abstract

We build a Taylor's expansion for composite functions. Some applications are introduced, where the proposed technique allows the authors to obtain an asymptotic expansion of high order in many small parameters of solutions.

## 1. Introduction

Let *f* ∈ *C* ^*N*+1^([0,1] × ℝ_+_ × ℝ^3^ × ℝ_+_^3^; ℝ) and *u*_*α*_ ∈ *W* = {*v* ∈ *L* ^*∞*^(0, *T*; *H* ^2^(0, 1)) : *v*_*t*_ ∈ *L* ^*∞*^(0, *T*; *H* ^1^(0,1))}, *α* ∈ *ℤ*_+_^*n*^, |*α*| ≤ *N*.

Let two functions *h*(*x*, *t*, *ε*) and  *F*(*x*, *t*, *ε*), which depend on (*x*, *t*) and *ε* = (*ε*_1_,…, *ε*_*n*_), be defined as follows:

(1) $$\begin{array}{c} h\left(x,t,\varepsilon \right)=\underset{\left|\alpha \right|\leq N}{\sum }u_{\alpha }\left(x,t\right)\varepsilon^{\alpha }, \end{array}$$

where

(2) $$\begin{array}{c} \varepsilon^{\alpha }=\varepsilon_{1}^{\alpha_{1}}\cdots \varepsilon_{n}^{\alpha_{n}},\;\;\varepsilon =\left(\varepsilon_{1},\ldots ,\varepsilon_{n}\right)\in {\mathbb{R}}^{n},\\ \alpha =\left(\alpha_{1},\ldots ,\alpha_{n}\right)\in {\mathbb{Z}}_{+}^{n},\\ \left|\alpha \right|=\alpha_{1}+\cdots +\alpha_{n},\\ F\left(x,t,\varepsilon \right)=f\left(x,t,h,h_{x},h_{t},{||h||}^{2},{||h_{x}||}^{2},{||h_{t}||}^{2}\right), \end{array}$$

where notation ||·|| stands for the norm in *L* ^2^(0,1).

The function *F*(*x*, *t*, *ε*) has the form of a composite function as follows:

(3) F(x,t,ε)=f(x,t,g1(ε),g2(ε),   g3(ε),g4(ε),g5(ε),g6(ε)),

where

(4) g1(ε)=h(x,t,ε)=∑|α|≤Nuα(x,t)εα,g2(ε)=hx(x,t,ε)=∑|α|≤Nuαx(x,t)εα,g3(ε)=ht(x,t,ε)=∑|α|≤Nuαt(x,t)εα,g4(ε)=||h(t,ε)||2=||∑|α|≤Nuα(t)εα||2=∑|δ|≤2N ∑|α|≤N ∑|β|≤N ∑α+β=δ〈uα(t),uβ(t)〉εδ=∑|α|≤2Ngα[4](t)εα,g5(ε)=||hx(t,ε)||2=||∑|α|≤Nuαx(t)εα||2=∑|δ|≤2N ∑|α|≤N ∑|β|≤N ∑α+β=δ〈uαx(t),uβx(t)〉εδ=∑|α|≤2Ngα[5](t)εα,g6(ε)=||ht(t,ε)||2=||∑|α|≤Nuαt(t)εα||2=∑|δ|≤2N ∑|α|≤N ∑|β|≤N ∑α+β=δ〈uαt(t),uβt(t)〉εδ=∑|α|≤2Ngα[6](t)εα,

where notation 〈·, ·〉 stands for the scalar product in *L* ^2^(0,1).

Under the above assumptions, in order to obtain an asymptotic expansion of high order in many small parameters of solutions in recent papers [1–4], we need to solve the following problem.

**Problem 1**

Establish the functions *F*_*α*_ = *F*_*α*_(*x*, *t*), |*α*| ≤ *N* (independent of *ε*) such that for $||\varepsilon ||=\sqrt{\varepsilon_{1}^{2}+\cdots +\varepsilon_{n}^{2}}$ being small enough,

(5) $$\begin{array}{c} F\left(x,t,\varepsilon \right)=\underset{\left|\alpha \right|\leq N}{\sum }F_{\alpha }\left(x,t\right)\varepsilon^{\alpha }+O\left({||\varepsilon ||}^{N+1}\right); \end{array}$$

it means that

(6) $$\begin{array}{c} {||F-\underset{\left|\alpha \right|\leq N}{\sum }F_{\alpha }\varepsilon^{\alpha }||}_{L^{\infty }(0,T,L^{2}(0,1))}\leq C_{N}{||\varepsilon ||}^{N+1}, \end{array}$$

where the constant *C*_*N*_ is independent from *ε*.

By Taylor's expansion for *F*(*x*, *t*, *ε*), it implies that

(7) $$\begin{array}{c} F_{\alpha }\left(x,t\right)=\frac{1}{\alpha !}D^{\alpha }F\left(x,t,0\right),\;\left|\alpha \right|\leq N. \end{array}$$

However, for *F*(*x*, *t*, *ε*) which is given in (3), it is very difficult to calculate *D* ^*α*^ *F*(*x*, *t*, 0), and so, the Problem 1 cannot proceed. Which  method can be used for solving the Problem 1? To answer, let us note that the function *F*(*x*, *t*, *ε*)  has the form of a composite function;  this view  suggests that  we need to construct the Taylor-Maclaurin expansion of the composite function. So, first in Section 2 we solve the following problem.

**Problem 2**

Let *Ω* be an open subset of ℝ^*n*^  and 0 ∈ *Ω*. Let  *g* = (*g*_1_,…, *g*_*p*_) ∈ *C* ^*N*+1^(*Ω*; ℝ^*p*^)  and  *f* ∈ *C* ^*N*+1^(ℝ^*p*^; ℝ).Seek the representation formula for *fog*, such that for ||*x*|| being small enough,

(8) (fog)(x)=f(g1(x),…,gp(x))=∑|α|≤Ndαxα+O(||x||N+1),

where *d*_*α*_, |*α*| ≤ *N* are calculated from the values of the given functions *f*; *g*_1_,…, *g*_*p*_  and of their derivatives at a suitable point.

Next in Section 3, as an application of the method used, we study the Problem 1. This technique is also a great help for the authors to obtain an asymptotic expansion as they want in recent papers [1–4].

## 2. Solving the Problem 2

We use the following notations. For a multi-index *α* = (*α*_1_,…, *α*_*n*_) ∈ *ℤ*_+_^*n*^ and *x* = (*x*_1_,…, *x*_*n*_) ∈ ℝ^*n*^, we put

(9) $$\begin{array}{c} \left|\alpha \right|=\alpha_{1}+\cdots +\alpha_{n},\;\;\;\alpha !=\alpha_{1}!\cdots \alpha_{n}!,\\ ||x||=\sqrt{x_{1}^{2}+\cdots +x_{n}^{2}},\;\;x^{\alpha }=x_{1}^{\alpha_{1}}\cdots x_{n}^{\alpha_{n}},\\ \alpha ,\beta \in {\mathbb{Z}}_{+}^{n},\;\alpha \leq \beta \Leftrightarrow \alpha_{i}\leq \beta_{i}\;\forall i=1,\ldots ,n. \end{array}$$

The following lemma is useful to solve the Problems 1 and 2.

**Lemma 3**

Let *m*, *N* ∈ *ℕ* and *a*_*α*_ ∈ ℝ, *α* ∈ *ℤ*_+_^*n*^, 1 ≤ |*α*| ≤ *N*. Then

(10) $$\begin{array}{c} {\left(\underset{1\leq \left|\alpha \right|\leq N}{\sum }a_{\alpha }x^{\alpha }\right)}^{m}=\underset{m\leq \left|\alpha \right|\leq mN}{\sum }T_{N}^{\left(m\right)}{\left[a\right]}_{\alpha }x^{\alpha }, \end{array}$$

where the coefficients *T*_*N*_^(*m*)^[*a*]_*α*_, *m* ≤ |*α*| ≤ *mN* depending on *a* = (*a*_*α*_), *α* ∈ *ℤ*_+_^*n*^, 1 ≤ |*α*| ≤ *N* are defined by the recurrent formulas

(11) TN(m)[a]α={uα,1≤|α|≤N,  m=1,∑β∈Aα(m)(N)aα−βTN(m−1)[u]β,m≤|α|≤mN,  m≥2,Aα(m)(N)={β∈ℤ+p:β≤α,1≤|α−β|≤N,       m−1≤|β|≤(m−1)N}.

The proof of Lemma 3 can be found in [2].

Now, using Taylor's expansion of the function *f*  around the point *g* = (*g*_1_,…, *g*_*p*_) ∈ ℝ^*p*^, we obtain that

(12) f(g1+H1,…,gp+Hp)  =f(g1,…,gp)   +∑1≤|α|≤N1α!Dαf(g1,…,gp)Hα   +RN[f,H],H=(H1,…,Hp)∈ℝp,  ||H||  is  small  enough,RN[f,H]=(N+1  ) ×∑|α|=N+11α!Hα∫01(1−θ)NDα         ×f(g+θH)dθ.

Similarly, we use Maclaurin's expansion of *g*_*i*_ as follows:

(13) Hi=gi(x)−gi(0)=∑1≤|β|≤N1β!Dβgi(0)xβ+RN[gi,x]=Pi+Ri, x∈ℝn, ||x||  is  small  enough,Pi=∑1≤|β|≤N1β!Dβgi(0)xβ,Ri=RN[gi,x]=(N+1)∑|β|=N+11β!xβ∫01(1−θ)NDβgi(θx)dθ.

Substituting (13) into (12), we get

(14) f(g1(x),…,gp(x))  =f(g1(0),…,gp(0))   +∑1≤|α|≤N1α!Dαf(g1(0),…,gp(0))Hα   +RN[f,H]  =f(g(0))+∑1≤|α|≤N1α!Dαf(g(0))Pα   +∑1≤|α|≤N1α!Dαf(g(0))(Hα−Pα)   +RN[f,H]  =f(g(0))+∑1≤|α|≤N1α!Dαf(g(0))Pα   +||x||N+1RN(1)[f,g,H,R],

where

(15) ||x||N+1RN(1)[f,g,H,R]  =∑1≤|α|≤N1α!Dαf(g(0))(Hα−Pα)+RN[f,H],RN[f,H]  =(N+1)∑|α|=N+11α!Hα∫01(1−θ)NDα               ×f(g(0)+θH)dθ.

Applying Lemma 3, with *a* = *σ* ^(*i*)^ = (*σ*_*β*_^(*i*)^), *σ*_*β*_^(*i*)^ = (1/*β*!)*D* ^*β*^ *g*_*i*_(0), 1 ≤ |*β*| ≤ *N*, it implies that

(16) Piαi=(∑1≤|β|≤N1β!Dβgi(0)xβ)αi≡(∑1≤|β|≤Nσβ(i)xβ)αi=∑αi≤|γi|≤αiNTN(α1)[σ(i)]γixγi.

Hence,

(17) Pα=P1α1⋯Ppαp=(∑1≤|β|≤N1β!Dβg1(0)xβ)α1 ⋯(∑1≤|β|≤N1β!Dβgp(0)xβ)αp=∑α1≤|γ1|≤α1NTN(α1)[σ(1)]γ1xγ1  ⋯∑αp≤|γp|≤αpNTN(αp)[σ(p)]γpxγp  =∑α1≤|γ1|≤α1N,⋮αp≤|γp|≤αpNTN(α1)[σ(1)]γ1⋯TN(αp)[σ(p)]γpxγ1+⋯+γp=∑α1≤|γ1|≤α1N,⋮αp≤|γp|≤αpNTN(α1)[σ(1)]γ1⋯TN(αp)[σ(p)]γpxδ=∑α1≤|γ1|≤α1N,⋮αp≤|γp|≤αpN∑γ1+⋯+γp=δTN(α1)[σ(1)]γ1  ⋯TN(αp)[σ(p)]γpxδ=∑|α|≤|δ|≤|α|N∑α1≤|γ1|≤α1N,⋮αp≤|γp|≤αpN∑γ1+⋯+γp=δTN(α1)[σ(1)]γ1  ⋯TN(αp)[σ(p)]γpxδ‍≡∑|α|≤|δ|≤|α|NΦδ[α,N,σ(1),…,σ(p)]xδ=∑|α|≤|δ|≤NΦδ[α,N,σ(1),…,σ(p)]xδ +∑N+1≤|δ|≤|α|NΦδ[α,N,σ(1),…,σ(p)]xδ,Φδ[α,N,σ(1),…,σ(p)]  =∑α1≤|γ1|≤α1N,⋮αp≤|γp|≤αpN∑γ1+⋯+γp=δTN(α1)[σ(1)]γ1    ⋯TN(αp)[σ(p)]γp.

Consequently,

(18) f(g(x))=f(g(0)) +∑1≤|α|≤N1α!Dαf(g(0))Pα +||x||N+1RN(1)[f,g,H,R]=f(g(0))+∑1≤|α|≤N1α!Dαf(g(0)) ×∑|α|≤|δ|≤|α|NΦδ[α,N,σ(1),…,σ(p)]xδ +||x||N+1RN(1)[f,g,H,R]  =f(g(0)) +∑1≤|α|≤N1α!Dαf(g(0)) ×∑|α|≤|δ|≤NΦδ[α,N,σ(1),…,σ(p)]xδ +∑1≤|α|≤N1α!Dαf(g(0)) ×∑N+1≤|δ|≤|α|NΦδ[α,N,σ(1),…,σ(p)]xδ +||x||N+1RN(1)[f,g,H,R]=f(g(0))+∑1≤|α|≤N1α!Dαf(g(0)) ×∑|α|≤|δ|≤NΦδ[α,N,σ(1),…,σ(p)]xδ   +||x||N+1RN(2)[f,g,H,R,x]=f(g(0)) +∑1≤|δ|≤N∑1≤|α|≤|δ|1α!Dαf(g(0))       ×Φδ[α,N,σ(1),…,σ(p)]xδ +||x||N+1RN(2)[f,g,H,R,x]=f(g(0))+∑1≤|δ|≤Ndδxδ +||x||N+1RN(2)[f,g,H,R,x].

Clearly, the Problem 2 is solved with

(19) $$\begin{array}{c} d_{\delta }=\sum_{1\leq |\alpha |\leq |\delta |}\frac{1}{\alpha !}D^{\alpha }f\left(g\left(0\right)\right)\Phi_{\delta }\left[\alpha ,N,\sigma^{\left(1\right)},\ldots ,\sigma^{\left(p\right)}\right]. \end{array}$$

## 3. Solving the Problem 1

 As an application of the method used in Section 2 for $p=6,\Omega =(0,1),\vec{\varepsilon }=(\varepsilon_{1},\ldots ,\varepsilon_{n})\in {\mathbb{R}}^{n}$, and *f* = *f*(*x*, *t*, *g*_1_,…, *g*_6_) ∈ *C* ^*N*+1^(ℝ^3^ × ℝ_+_^3^; ℝ), for each  fixed (*x*, *t*) ∈ [0,1] × ℝ_+_; *g*_*i*_ ∈ *C* ^*N*+1^(*Ω*; ℝ), *i* = 1,2, 3, for each  fixed (*x*, *t*) ∈ [0,1] × ℝ_+_ : *g*_*i*_ ∈ *C* ^*N*+1^(*Ω*; ℝ_+_), *i* = 4,5, 6, for each  fixed *t* ∈ ℝ_+_, we now investigate the Problem 1.

For each  fixed (*x*, *t*) ∈ [0,1] × ℝ_+_, using Taylor's expansion of the function *f* around the point *g* = (*g*_1_,…, *g*_6_) ∈ ℝ^3^ × ℝ_+_^3^ up to order *N*, we obtain that

(20) f(x,t,g+H)  =f(x,t,g1+H1,…,g6+H6)  =f(x,t,g1,…,g6)   +∑1≤|α|≤N1α!Dαf(x,t,g1,…,g6)Hα   +||H||N+1RN(1)[f,H],

(21) ||H||N+1RN(1)[f,H]  =(N+1)∑|α|=N+11α!Hα∫01(1−θ)NDα               ×f(g+θH)dθ,

where *H* = (*H*_1_,…, *H*_6_) ∈ ℝ^6^, ||*H*|| is small enough, *D*_*i*_^*α*_*i*_^ *f* = ∂*f*/∂*g*_*i*_, *i* = 1,2,…, 6.

Next, the precise structure of the representation formulas for *g*_1_,…, *g*_6_  will be needed below to continue.

For each fixed (*x*, *t*) ∈ [0,1] × ℝ_+_: we have the following.

(i) The representation formula for *g*_*i*_(*ε*) = *g*_*i*_(*x*, *t*, *ε*), *i* = 1,2, 3.

We rewrite *g*_1_(*ε*) as follows

(22) g1(ε)=∑|α|≤Nuα(x,t)εα≡g1(0)+∑1≤|β|≤Ngβ[1](x,t)εβ≡g1(0)+H1,

in which

(23) $$\begin{array}{c} g_{1}\left(0\right)=g_{1}\left(x,t\right)=u_{0}\left(x,t\right),\;\left|\beta \right|=0,\\ g_{\beta }^{\left[1\right]}\left(x,t\right)=u_{\beta }\left(x,t\right),\;1\leq \left|\beta \right|\leq N. \end{array}$$

It is similar to *g*_2_(*ε*), *g*_3_(*ε*); we write

(24) g2(ε)=∑|α|≤Nuαx(x,t)εα≡g2(0)+∑1≤|β|≤Ngβ[2](x,t)εβ≡g2(0)+H2,g3(ε)=∑|α|≤Nuαt(x,t)εα≡g3(0)+∑1≤|β|≤Ngβ[3](x,t)εβ≡g3(0)+H3,

where

(25) $$\begin{array}{c} g_{2}\left(0\right)=g_{2}\left(x,t\right)=u_{0x}\left(x,t\right),\\ g_{3}\left(0\right)=g_{3}\left(x,t\right)=u_{0t}\left(x,t\right),\;\left|\beta \right|=0,\\ g_{\beta }^{\left[2\right]}\left(x,t\right)=u_{\beta x}\left(x,t\right),\\ g_{\beta }^{\left[3\right]}\left(x,t\right)=u_{\beta t}\left(x,t\right),\;1\leq \left|\beta \right|\leq N. \end{array}$$

For each  fixed *t* ∈ ℝ_+_: we have the following.

(ii) The representation formula for *g*_*i*_(*ε*) = *g*_*i*_(*t*, *ε*), *i* = 4,5, 6.

Write

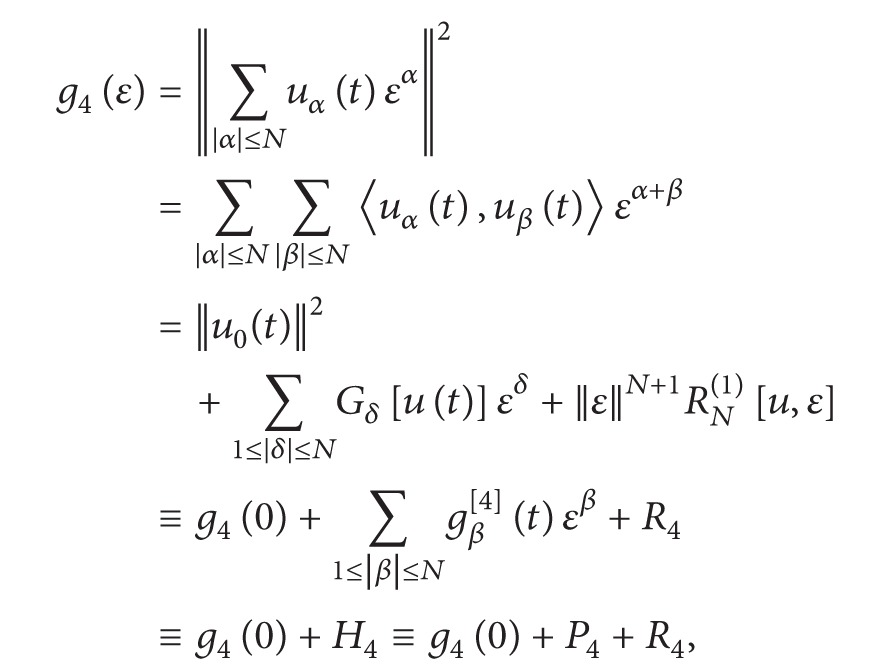
(26)

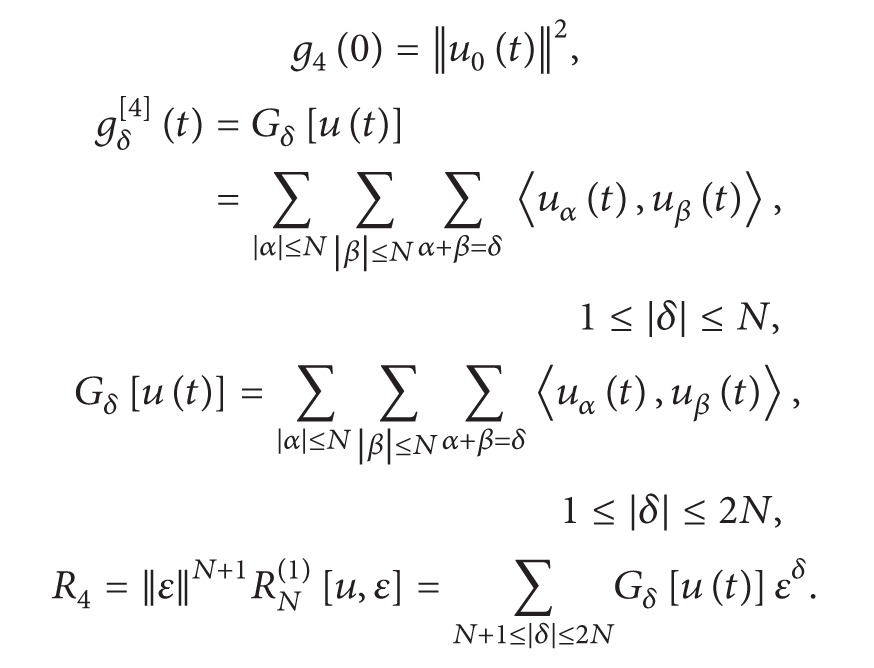
(27)


Similarly,

(28) g5(ε)=||∑|α|≤Nuαx(t)εα||2=∑|α|≤N ∑|β|≤N〈uαx(t),uβx(t)〉εα+β=||u0x(t)||2+∑1≤|δ|≤NGδ[ux(t)]εδ +||ε||N+1RN(1)[ux,ε]≡g5(0)+∑1≤|β|≤Ngβ[5](t)εβ+R5≡g5(0)+H5≡g5(0)+P5+R5,

in which

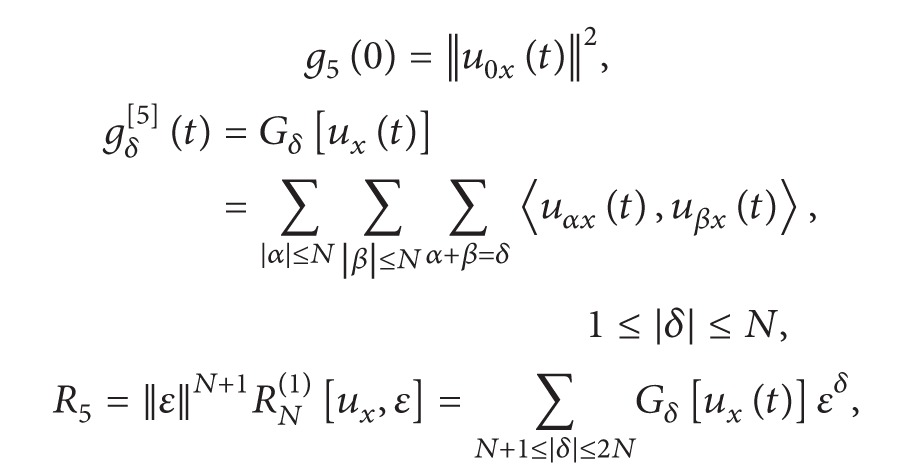
(29)

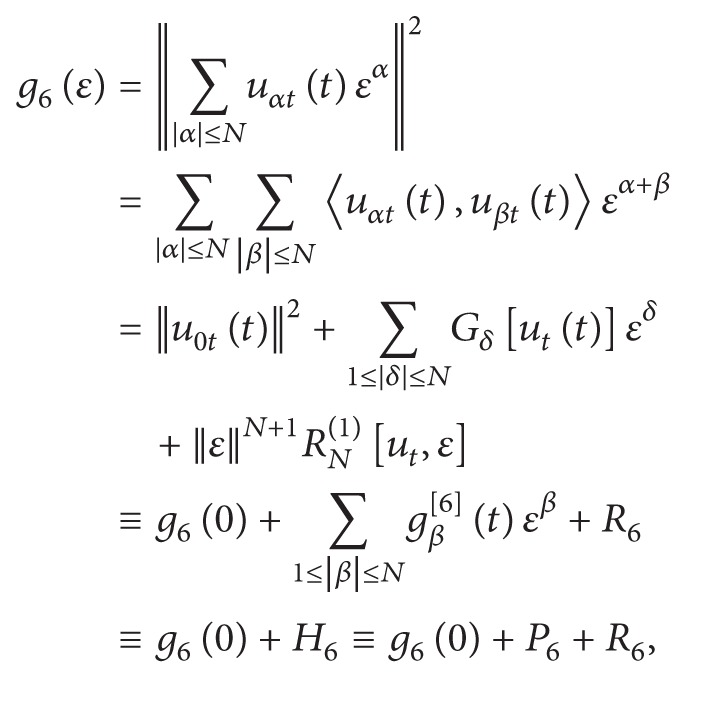
(30)
where

(31) g6(0)=||u0t(t)||2,gδ[6](t)=Gδ[ut(t)]=∑|α|≤N ∑|β|≤N ∑α+β=δ〈uαt(t),uβt(t)〉,           1≤|δ|≤N,R6=||ε||N+1RN(1)[ut,ε]=∑N+1≤|δ|≤2NGδ[ut(t)]εδ.

Then  (22), (24), (26), (28), and (30) imply that

(32) $$\begin{array}{c} g_{i}\left(\varepsilon \right)-g_{i}\left(0\right)=H_{i}=P_{i}+R_{i}=\underset{1\leq \left|\beta \right|\leq N}{\sum }g_{\beta }^{\left[i\right]}\varepsilon^{\beta }+R_{i},\\ R_{i}=0,\;i=1,2,3,\;\;R_{j}=O\left({||\varepsilon ||}^{N+1}\right),\;j=4,5,6. \end{array}$$

We also need the following lemma.

**Lemma 4**

For all *α* = (*α*_1_,…, *α*_*n*_) ∈ *ℤ*_+_^*n*^, |*α* | ≥ 1, then

(33) Hα=H1α1H2α2H3α3H4α4H5α5H6α6=∑|α|≤|δ|≤NΦδ[α,N,σ(1),…,σ(6)]εδ+O(||ε||N+1),

where

(34) Φδ[α,N,σ(1),…,σ(6)] =∑α1≤|γ1|≤α1N,⋮α6≤|γ6|≤α6N∑γ1+⋯+γ6=δTN(α1)[σ(1)]γ1⋯TN(α6)[σ(6)]γ6,σ(i)=(σβ(i)),  σβ(i)=gβ[i], 1≤|β|≤N,  i=1,2,…,6.

**Proof of Lemma 4**

We have

(35) Hα=H1α1H2α2H3α3H4α4H5α5H6α6=P1α1P2α2P3α3(P4+R4)α4(P5+R5)α5(P6+R6)α6=P1α1P2α2P3α3P4α4P5α5P6α6+P1α1P2α2P3α3 ×[(P4+R4)α4(P5+R5)α5(P6+R6)α6−P4α4P5α5P6α6].

Note that *R*_*j*_ = *O*(||*ε*||^*N*+1^), *j* = 4,5, 6, so

(36) (P4+R4)α4(P5+R5)α5(P6+R6)α6−P4α4P5α5P6α6 =(P4+R4)α4(P5+R5)α5(P6+R6)α6  −P4α4(P5+R5)α5(P6+R6)α6  +P4α4(P5+R5)α5(P6+R6)α6−P4α4P5α5(P6+R6)α6  +P4α4P5α5(P6+R6)α6−P4α4P5α5P6α6 =[(P4+R4)α4−P4α4](P5+R5)α5(P6+R6)α6  +P4α4[(P5+R5)α5−P5α5](P6+R6)α6  +P4α4P5α5[(P6+R6)α6−P6α6] =R4[∑j=0α4−1(P4+R4)jP4α4−1−j]  ×(P5+R5)α5(P6+R6)α6  +R5[∑j=0α5−1(P5+R5)jP5α5−1−j]P4α4(P6+R6)α6  +R6[∑j=0α6−1(P6+R6)jP6α6−1−j]P4α4P5α5=||ε||N+1RN(1)[H,α,ε]=O(||ε||N+1).

Combining (35) and (36) yields

(37) Hα=H1α1H2α2H3α3H4α4H5α5H6α6=P1α1P2α2P3α3(P4+R4)α4(P5+R5)α5(P6+R6)α6=P1α1P2α2P3α3P4α4P5α5P6α6 +||ε||N+1RN(1)[H,α,ε]=(∑1≤|β|≤Ngβ(1)εβ)α1⋯(∑1≤|β|≤Ngβ(6)εβ)α6 +||ε||N+1RN(1)[H,α,ε]=∑|α|≤|δ|≤NΦδ[α,N,σ(1),…,σ(6)]εδ +∑N+1≤|δ|≤|α|NΦδ[α,N,σ(1),…,σ(6)]εδ +||ε||N+1RN(1)[H,α,ε]≡∑|α|≤|δ|≤NΦδ[α,N,σ(1),…,σ(6)]εδ +||ε||N+1RN(2)[H,α,ε],

where

(38) ||ε||N+1RN(2)[H,α,ε]  =∑N+1≤|δ|≤|α|NΦδ[α,N,σ(1),…,σ(6)]εδ   +||ε||N+1RN(1)[H,α,ε].

Lemma 4 is proved.

Substituting (37) into (20), we obtain

(39) F(x,t,εα) =f(x,t,h,hx,ht,||h||2,||hx||2,||ht||2) =f(x,t,g+H)=f(x,t,g(0)+H) =f(x,t,g1(0)+H1,…,g6(0)+H6) =f(x,t,g(0))  +∑1≤|α|≤N1α!Dαf(x,t,g(0))Hα+||H||N+1RN(1)[f,H] =f(x,t,g(0))  +∑1≤|α|≤N1α!Dαf(x,t,g(0))      ×  [∑|α|≤|δ|≤NΦδ[α,N,σ(1),…,σ(6)]εδ        + ||ε||N+1RN(2)[H,α,ε]]  +||H||N+1RN(1)[f,H] =f(x,t,g(0))  +∑1≤|α|≤N1α!Dαf(x,t,g(0))      ×[∑|α|≤|δ|≤NΦδ[α,N,σ(1),…,σ(6)]εδ]  +||ε||N+1∑1≤|α|≤N1α!Dαf(x,t,g(0))RN(2)[H,α,ε]          +||H||N+1RN(1)[f,H] =f(x,t,g(0))  +∑1≤|α|≤N1α!Dαf(x,t,g(0))      ×∑|α|≤|δ|≤NΦδ[α,N,σ(1),…,σ(6)]εδ  +||ε||N+1RN(3)[f,g,H,ε] =f(x,t,g(0))  +∑1≤|δ|≤N∑|α|≤|δ|1α!Dαf(x,t,g(0))Φδ        ×[α,N,σ(1),…,σ(6)]εδ  +||ε||N+1RN(3)[f,g,H,ε→] =f(x,t,g(0))+∑1≤|δ|≤NFδ(x,t)εδ  +||ε||N+1RN(3)[f,g,H,ε],

where

(40) ||ε||N+1RN(3)[f,g,H,ε]=||ε||N+1∑1≤|α|≤N1α!Dαf(x,t,g(0))RN(2)[H,α,ε] +||H||N+1RN(1)[f,H],  Fδ(x,t)=∑|α|≤|δ|1α!Dαf(x,t,g(0))Φδ[α,N,σ(1),…,σ(6)],               δ∈ℤ+6,1≤|δ|≤N.

Since *u*_*α*_ ∈ *W* = {*v* ∈ *L* ^*∞*^(0, *T*, *H* ^2^(0,1)) : *v*_*t*_ ∈ *L* ^*∞*^(0, *T*, *H* ^1^(0,1))}, *α* ∈ *ℤ*_+_^*n*^, |*α*| ≤ *N*, hence *u*_*α*_, *u*_*αx*_, *u*_*αt*_ ∈ *L* ^*∞*^(0, *T*, *H* ^1^(0,1)) ⊂ *L* ^*∞*^(*Q*_*T*_). Then from (39) and (40)_1_, we get

(41) ||F(ε)−∑|δ|≤NFδεδ||L∞(0,T,L2(0,1)) =||ε||N+1||RN(3)[f,g,H,ε]||L∞(0,T,L2(0,1))≤CN||ε||N+1,

for ||*ε*|| is small enough; consequently the Problem 1 is solved.

**Remark 5**

(i) This result improves the one in [1], for *n* = 1.

(ii) In case of *F*(*x*, *t*, *ε*) = *μ*(*x*, *t*, *h*, ||*h*_*x*_||^2^), where *μ* ∈ *C* ^*N*+1^([0,1] × ℝ_+_ × ℝ × ℝ_+_; ℝ), *n* = *p*, this result is also obtained in [3].
